# Severe respiratory distress in term infants born electively at high altitude

**DOI:** 10.1186/1471-2393-6-4

**Published:** 2006-02-16

**Authors:** Ahmad F Bakr, Mohammad M Abbas

**Affiliations:** 1 Departments of Pediatrics, University of Alexandria, Alexandria, Egypt; 2 Departments of Obstetrics, King Abdul-Aziz Specialist Hospital, Taif, Saudi Arabia; 3 Consultant Neonatologist, Welcare Hospital, PO Box 31500 Dubai, United Arab Emirates; 4 Obstetrics and Gynecology, Faculty of Medicine, University of Alexandria, Egypt

## Abstract

**Background:**

We studied the contribution of elective delivery to severe respiratory distress syndrome (RDS) in term babies born at high altitude.

**Methods:**

We prospectively studied the charts of term babies born in Taif Maternity Hospital (1640 m above sea level) between 1/1/2004 and 31/10/2004 who developed RDS and required mechanical ventilation.

**Results:**

8634 deliveries occurred from 37–<41 weeks; 13 (0.15%) had RDS requiring mechanical ventilation. Seven infants delivered at 37–<38 weeks, (OR for RDS = 26 95%CI -4.6 to 5.8), five delivered at 38–<39 weeks, (OR for RDS = 10 95%CI -4.9 to 5.4) and one delivered at >39 weeks. Six of 13 infants were electively delivered without documented lung maturity.

**Conclusion:**

Infants born at 37 and 38 weeks' gestation remain at significantly increased risk for severe RDS. Elective delivery is responsible for 50% of the potentially avoidable cases. Our data suggest that the altitude does not seem to influence the incidence of severe RDS in term infants born electively.

## Background

Respiratory distress syndrome (RDS) is a common problem in neonatal practice, usually seen in premature babies. Iatrogenic RDS is defined as RDS resulting from elective delivery [1]. Worldwide, iatrogenic RDS persists as a cause of neonatal morbidity and mortality [2,3]. Non-adherence to the guidelines [4] designed to minimize the risk of electively delivering an immature infant can lead to iatrogenic RDS. This problem is further augmented in developing countries where good antenatal care is not always the rule. Not knowing the expected dates of delivery accurately by the patient and/or the treating physician is not uncommon. Further contributing to the problem is the variable definition of a term pregnancy. Fetal lung maturity cannot be assumed with certainty until 39 weeks' gestation [5]. The concept of maturity is even more different in some communities, including the educated, in developing countries.

Obstetric practice in Taif is peculiar. The incidence of high gravidity and parity is common and elective sections tend to be done earlier for fear of scar dehiscence. Also Taif lies at high altitude; 1640 m above sea level. High altitude implies a decrease in the atmospheric pressure and consequently oxygenation. It is known to cause intrauterine growth restriction (IUGR), which raises neonatal mortality. However, existing studies are unclear as to whether IUGR-specific mortality at high altitude is similar to, less than, or greater than at low altitude [6,7]. Whether this is associated with a protective beneficial effect on lung maturity or not is not clear. Whether this should change or not the guidelines for elective delivery is also not clear and has to be studied.

We planned to determine the incidence of severe RDS requiring mechanical ventilation at term among those delivered electively at high altitude.

## Methods

This study was carried-out in the maternity section of King Abul-Aziz Specialist Hospital in Taif (1640 m above sea level) between 1/1/2004 and 31/10/2004. The study was approved by KAASH ethical committee. Taif Maternity Hospital is a large urban institution with approximately 12,000 deliveries per year. All newborns delivered at 37 weeks' gestation or more and having severe RDS requiring mechanical ventilation were enrolled in the study. Exclusion criteria included any cause of respiratory distress apart from RDS (sepsis, pneumonia, meconium aspiration, asphyxia, pulmonary hemorrhage and congenital abnormalities).

The diagnosis of RDS was made by the NICU staff. Every patient record was reviewed by a neonatologist to confirm the diagnosis and to ensure the absence of exclusion criteria. RDS was diagnosed by the classic clinical and radiological signs of respiratory distress syndrome. Oxygen and ventilator requirements were recorded. The mothers' files were checked to confirm gestational age and to identify the reason for delivery. Deliveries were classified as spontaneous (mother presented in labor), indicated (obstetric or medical reasons), and elective (mother not in labor and no maternal or fetal condition warranting delivery). The differences in outcomes in relation to gestational age were analyzed. Comparisons with figures at sea level were done when applicable. Paired t-test was performed, p values < 0.05 were considered significant. Odds ratio and 95% confidence intervals were calculated..5

## Results

During the study period, 8634 infants were delivered between 37 and 41 weeks' gestation. Of these children, 1338 (15.5%) delivered at 37 – <38, 2478 (28.7%) at 38 – <39, and 4818 (55.8%) at 39 – <41 weeks. Thirteen (0.15%) of these infants experienced RDS requiring mechanical ventilation.

The maternal and neonatal demographic data were not significantly different at different gestational ages (Table 1). None of the mothers of the index cases received antenatal steroids.

**Table 1:** Maternal and Neonatal Characteristics of Index Cases (Mean ± SD)

	**Gestational Age at Delivery**		
	
	37 – <38 (n= 7)	38 – <39 (n= 5)	39 – ≤41 (n= 1)
**Maternal Data**			
• Age (years)	30.8 ± 4.2	29.3 ± 5.8	30.7
• Parity			
0	1	1	0
1	2	1	1
≥ 2	4	3	1
• Cesarean Delivery	2	3	1
**Newborn Data**			
• Birth weight (g)	2770 ± 425	3070 ± 350	3140
• 5-min Apgar < 7	1	0	0
• Ventilator Days	4.6 ± 2	3.7 ± 2.1	3
• Surfactant given	5	3	0
• Hospital Stay (days)	12.4 ± 3.7	11.7 ± 3.8	11
• Air Leak	2	1	1
• Deaths	1	1	0

Seven of the affected infants were delivered at 37–38 weeks (5.2/1000 births) (OR = 26, 95%CI -4.6 to 5.8). Five patients delivered at 38–39 weeks, (2/1000 births) (OR = 10 95%CI -4.9 to 5.4). One infant was born between 39–41 weeks, (0.2 per 1000 births) [Comparison group]. None of these children developed chronic lung disease.

Out of the thirteen infants, six (46.15%) were delivered electively. All of the six neonates were born at <39 weeks' gestation without an assessment of fetal lung maturity. (Table 2). Further complications including air leaks or pulmonary hypertension were reported in four out of the six (66.7%). The frequency of RDS requiring mechanical ventilation following elective delivery was not different than that observed after indicated or spontaneous delivery, either overall or when stratified by gestational age.

**Table 2:** Data of Infants with iatrogenic RDS

**Case**	**Gestation Age **(Wks + D)*	**Birth Weight **(g)	**Ventilator Days**	**Elective Delivery**
1	38 + 1	3450	4	Planned repeat CS
2	37 + 3	3050	2	Breech, not in labor
3	38	3700	5	Planned repeat CS
4	38 + 4	3200	2	Planned repeat CS
5	37	3300	1	Planned repeat CS
6	38 + 2	3750	11	Planned repeat CS

* Calculated from first day of last menstrual period

## Discussion

In our series, we found that infants born at 37 and 38 weeks' gestation experience a 26-fold and 10-fold increased risk of severe RDS, respectively, as compared with 39 to 41 weeks' newborns. Our observed rates of RDS stratified by weeks of gestation are comparable to those reported by Madar et al [8]. Two studies reported higher rates of term RDS. The methodology of the first one was similar to ours [2]. However the study of Bowers et al [9] did not require the use of positive pressure ventilation in diagnosing RDS. Their higher rates more likely reflected the inclusion of the more common mild cases of neonatal respiratory distress.

The primary objective of this study was to assess the contribution of elective delivery to severe neonatal RDS. In our study, this practice was responsible for 46% of severe RDS at 37 and 38 weeks' gestation. Bowers et al [9] reported in 1982 that over 33% of newborn RDS was iatrogenic. Madar et al [8] concluded that babies who are not premature, using the internationally agreed definition, can show signs of potentially lethal pulmonary immaturity at birth, especially if subjected to pre-labour caesarean delivery. Those born at 37–38 wk are 120 times more likely to receive ventilatory support for surfactant deficiency than those born at 39–41 wk. Later on, the reports varied significantly [10]. Zanardo et al stated that infants born by elective caesarean delivery at term are at increased risk for developing respiratory disorders compared with those born by vaginal delivery. A significant reduction in neonatal RDS would be obtained if elective caesarean delivery were performed after 39 + 0 gestational weeks of pregnancy [11].

Cesarean delivery, especially when performed before the onset of labor, incurs additional risk of neonatal respiratory complications [12-14]. This risk is attributable to pulmonary immaturity, and the lack of the beneficial effects of normal labor on the newborn. These effects include reduction in lung water, enhanced cathecholamine levels, secretion of surfactant stores into the alveolar space, and increased levels of pulmonary vasodilating substances [15-19]. In our series, all of the electively delivered patients whose infants experienced RDS underwent scheduled cesarean delivery before labor and without assessment of lung maturity. This findings is consistent with other studies noting that 60–100% of electively delivered infants with RDS were born by cesarean [20].

Neonatal morbidity in term infants with RDS requiring mechanical ventilation was severe. Nearly thirty per cent had pulmonary air leak, most of them were electively delivered. This coincides with the findings of other studies that reported air leaks in 34%, pulmonary hypertension in 20% and requiring ECMO in 16% of infants with RDS following elective delivery [10,13].

Deaths occurred in 15% in our series, while it is none in most of the other reports. The two deaths in our series were among those electively delivered before 39 weeks. In fact, the high mortality rate among newborn babies undergoing mechanical ventilation is high in developing countries regardless the indication of ventilation. The RDS, which necessitated mechanical ventilation, is related to the morbidity of these newborns, while the high mortality rate is more directly related to the level of neonatal intensive care in developing countries. Testing for fetal lung maturity is not routinely practiced in developing countries.

Many studies looked at the effect of high altitudes on neonatal outcome.[21,22] However, none studied the incidence of iatrogenic RDS in such environment. It was well-shown that the incidence of IUGR is higher in pregnancies at high altitudes [6,7]. In our study, the average birth weights seemed lower than those normally recorded at comparable gestational ages. However, we did not have lower incidence of term RDS in our series compared to other studies done at sea level [2,3,8,11]. One of the limitations of our study was that we did not compare our results with a similar centre at sea level. Another limitation is that the number of term infants who had severe RDS is small to draw solid conclusions with regards to the relation with high altitude. Further long term studies involving larger numbers of cases and those done simultaneously at different altitudes can draw better conclusions.

Iatrogenic RDS may result from misinterpreting clinical or sonographic data, nonadherence to clinical protocols, or failing to perform amniocentesis to assess fetal lung maturity. Strategies to minimize the chance of electively delivering an immature fetus include awaiting onset of spontaneous labor (when applicable) before performing cesarean delivery, appropriate interpretation of clinical dating and ultrasound parameters, or obtaining amniotic fluid lung maturity studies [1-4]. In our study, none of the infants was tested for lung maturity. Adherence to the recommended guidelines may have avoided most of the morbidities.

## Conclusion and recommendations

Infants born at 37 and 38 weeks' gestation remain at significantly increased risk for severe RDS. Fifty per cent of severe RDS cases at these gestational ages followed elective delivery without prior assessment of fetal lung maturity, and were potentially avoidable. Adherence to clinical protocols should minimize the frequency with which severe RDS occurs in term newborn. In comparison with previous studies made at sea level, our data suggest that the altitude does not seem to influence the incidence of severe RDS in term infants born electively. Further well-planned multicenter studies are needed.

## Competing interests

The author(s) declare that they have no competing interests.

## Authors' contributions

AB carried out the study design, data collection, analysis and writing of the manuscript. MA shared participated in the design of the study and shared in the data collection. All authors read and approved the final manuscript.

## Pre-publication history

The pre-publication history for this paper can be accessed here:


